# Engineering isoprenoids production in metabolically versatile microbial host *Pseudomonas putida*

**DOI:** 10.1186/s13068-022-02235-6

**Published:** 2022-12-12

**Authors:** Xi Wang, Edward E. K. Baidoo, Ramu Kakumanu, Silvia Xie, Aindrila Mukhopadhyay, Taek Soon Lee

**Affiliations:** 1 Joint BioEnergy Institute (JBEI), 5885 Hollis St., Emeryville, CA 94608 USA; 2 Biological Systems & Engineering Division, Lawrence Berkeley National Laboratory, Berkeley, CA 94720 USA; 3 Department of Molecular & Cell Biology, University of California, Berkeley, CA 94720 USA

**Keywords:** *P. putida* KT2440, Isoprenol, Mevalonate, IPP-bypass pathway, l-Glutamate, *p*-Coumarate, Isoprenoid

## Abstract

With the increasing need for microbial bioproduction to replace petrochemicals, it is critical to develop a new industrial microbial workhorse that improves the conversion of lignocellulosic carbon to biofuels and bioproducts in an economically feasible manner. *Pseudomonas putida* KT2440 is a promising microbial host due to its capability to grow on a broad range of carbon sources and its high tolerance to xenobiotics. In this study, we engineered *P. putida* KT2440 to produce isoprenoids, a vast category of compounds that provide routes to many petrochemical replacements. A heterologous mevalonate (MVA) pathway was engineered to produce potential biofuels isoprenol (C_5_) and epi-isozizaene (C_15_) for the first time in *P. putida*. We compared the difference between three different isoprenoid pathways in *P. putida* on isoprenol production and achieved 104 mg/L of isoprenol production in a batch flask experiment through optimization of the strain. As *P. putida* can natively consume isoprenol, we investigated how to prevent this self-consumption. We discovered that supplementing l-glutamate in the medium can effectively prevent isoprenol consumption in *P. putida* and metabolomics analysis showed an insufficient energy availability and an imbalanced redox status during isoprenol degradation. We also showed that the engineered *P. putida* strain can produce isoprenol using aromatic substrates such as *p*-coumarate as the sole carbon source, and this result demonstrates that *P. putida* is a valuable microbial chassis for isoprenoids to achieve sustainable biofuel production from lignocellulosic biomass.

**Graphical Abstract:**

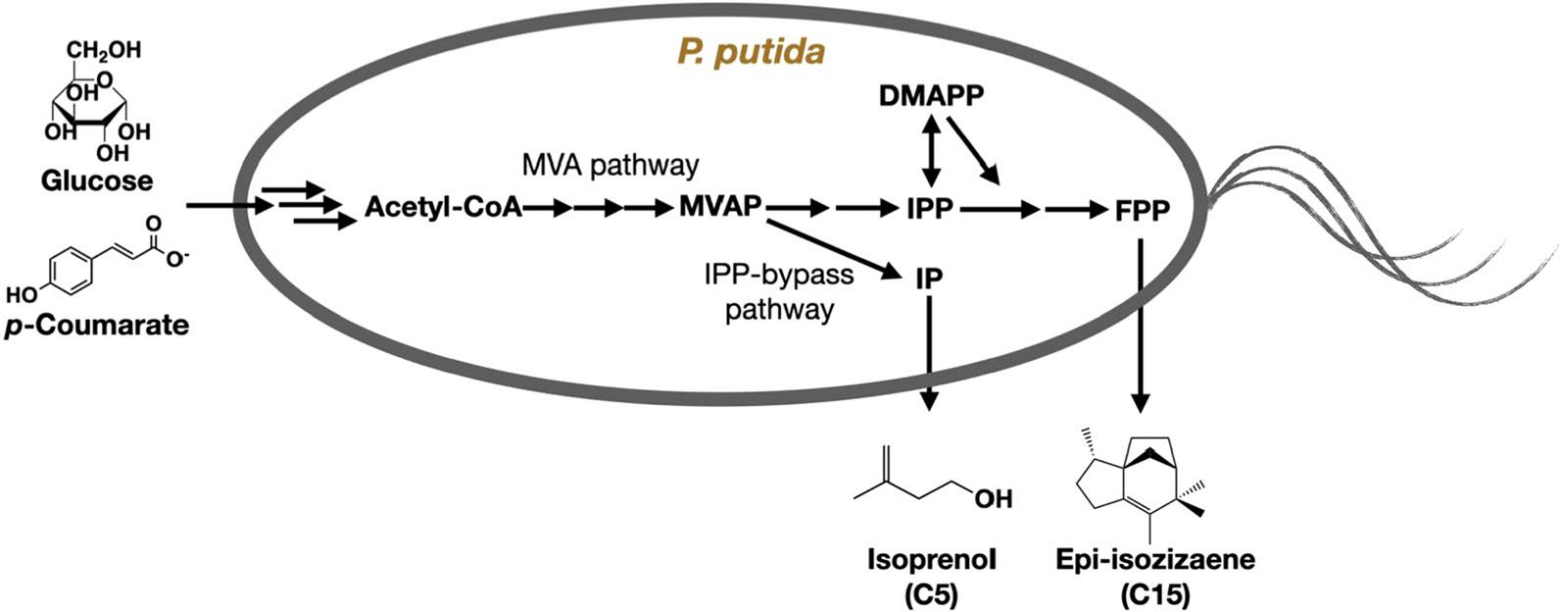

**Supplementary Information:**

The online version contains supplementary material available at 10.1186/s13068-022-02235-6.

## Introduction

Increasing concerns of climate change and energy security have necessitated microbial biosynthesis to produce biofuels from renewable carbon source as a sustainable and stable alternative to the fossil fuel-based approaches [1, 2]. Biofuels research to date has predominantly focused on conversion of sugars (hexoses and pentoses), the primary components of deconstructed lignocellulosic biomass [3]. However, lignin is another major component of lignocellulosic biomass and its catabolism has also been extensively studied recently [4–6]. Development of a new microbial chassis that enables full utilization of the lignocellulosic biomass-derived carbon sources is critical to achieve economically viable biofuel production [7].

**Fig. 1:**
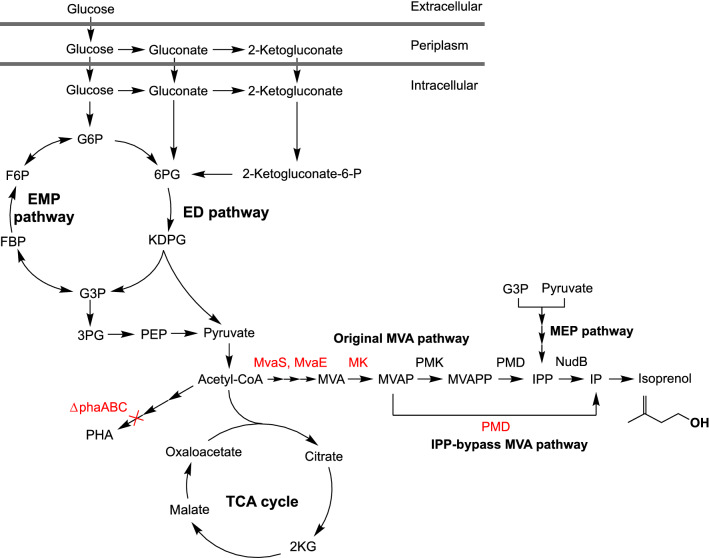
Central metabolism and isoprenol production pathways in *P. putida*. Isoprenol production pathways, including the MEP pathway, the original MVA pathway, and the IPP-bypass MVA pathway are presented and the key engineering efforts to overexpress or knockout genes are presented in red. G6P, glucose-6-phosphate; F6P, fructose-6-phosphate; FBP, fructose-1,6-diphosphate; G3P, glyceraldehyde-3-phosphate; 3PG, 3-phosphoglycerate; PEP, phosphoenolpyruvate; 2 KG, 2-ketoglutarate; MVA, mevalonate; MVAP, mevalonate phosphate; MVAPP, mevalonate diphosphate; IPP, isopentenyl diphosphate; DMAPP, dimethylallyl diphosphate; IP, isopentenyl phosphate; PHA, polyhydroxyalkanoate; MK, mevalonate kinase; PMK, phosphomevalonate kinase; PMD, phosphomevalonate decarboxylase; NudB, dihydroneopterin triphosphate diphosphatase. MvaES, HMGS and HMGR genes from *Enterococcus faecalis*; phaABC, PHA synthase

*Pseudomonas putida* KT2440 has recently emerged as a promising microbial host due to its capability of utilizing a broad range of carbon sources and its high tolerance to xenobiotics [8]. As *P. putida* is usually isolated from soils [9], the natural living environment conveys to *P. putida* versatile metabolism to degrade different types of substrates as carbon sources and it is adapted to tolerate various physicochemical stresses. Particularly, *P. putida* can utilize lignin-derived intermediates and aromatics, such as *p*-coumarate, benzoate, toluene as sole carbon sources, and thus has great potential to be developed as a new microbial workhorse to convert renewable carbon sources during bio-based production. *P. putida* KT2440 has been generally recognized as safe (GRAS) and is widely used for metabolic engineering studies as its full genome sequence is available [10]. It can share some genetic parts (plasmid backbone, promoter, RBS, etc.) with *Escherichia coli*, which could facilitate the genetic modification in *P. putida*. However, *P. putida* also showed different sugar metabolism from the model hosts that use classic glycolysis pathway, such as *E. coli*, *Saccharomyces cerevisiae*. *P. putida* oxidizes glucose to gluconate and 2-ketogluconate in the periplasm, followed by the phosphorylation to 6-phophogluconate (6PG) toward Entner–Doudoroff (ED) pathway [11] (Fig. 1). Due to the lack of phosphofructokinase (PFK) that catalyzes the rate-limiting phosphorylation of fructose-6-phosphate (F6P) to fructose-1,6-diphosphate (FBP) in glycolysis, *P. putida* does not catabolize glucose through the typical glycolysis but by the ED pathway [11].

Microbial production of isoprenoids has been considered a critical route for developing biofuels [12]. The biosynthesis of isoprenoids starts with two key isoprene units, isopentenyl diphosphate (IPP) and dimethylallyl diphosphate (DMAPP), naturally synthesized by two isoprenoid pathways: the mevalonate (MVA) pathway and non-mevalonate (methylerythritol 4-phosphate, MEP) pathway [13], respectively. IPP and DMAPP are precursors of isopentenols (C_5_) [14], and they can also be condensed to geranyl diphosphate (GPP) and farnesyl diphosphate (FPP) to produce monoterpenes (C_10_) [15, 16] and sesquiterpenes (C_15_) [17, 18], respectively. All C_5_, C_10_, and C_15_ isoprenoids are important candidates for fuel, especially jet fuel replacements [19]. Typical isoprenoid fuel molecules include isoprenol (C_5_) [20], limonene (C_10_) [15], 1,8-cineole (C_10_) [16], bisabolene (C_15_) [17], epi-isozizaene (C_15_) [18], etc. Among them, isoprenol (3-methyl-3-buten-1-ol) has received more attention due to its increasing applications as a valuable drop-in fuel molecule and important precursor of commodity chemicals. For example, US Navy has recently developed a high-performance jet fuel, 1,4-dimethylcyclooctane (DMCO), which can be produced from isoprenol [21]. Isoprenol is the dephosphorylated molecule of isopentenyl phosphate (IP) [22]. Unlike monoterpenes and sesquiterpenes, isoprenol synthesis does not require IPP which is toxic to cell growth [23]. An IPP-bypass pathway was developed to overcome the IPP toxicity and showed advantages in isoprenol (C_5_) production in both *E. coli* [24] and yeast [20].

Given that *P. putida* has emerged as a new workhorse strain, it has attracted interests for engineering of isoprenoid production [25–27]. For example, there have been a few literatures for isoprenoids production in *P. putida*, and mostly, the endogenous MEP pathway was engineered for the isoprenoids production and frequently focused on the oxidation of terpenes using P450 enzymes as *P. putida* is known to be tolerant to oxidative stress [25]. The heterologous MVA pathway was also expressed in *P. putida*, but the performance was not as good as what was shown in *E. coli* when a similar engineering strategy was attempted, and only a low productivity and titers were achieved [26].

In this study, we engineered the heterologous MVA pathway in *P. putida* KT2440 to produce isoprenoids, including isoprenol (C_5_) and epi-isozizaene (C_15_). We compared the differences among the MEP, MVA, and IPP-bypass MVA pathways during isoprenol production (Fig. 1). Since isoprenol can be utilized as a carbon source by *P. putida* KT2440 [28], we investigated strategies to prevent isoprenol self-consumption. Metabolomics was performed to reveal the metabolic difference during isoprenol degradation. We also showed the engineered *P. putida* can produce isoprenol using *p*-coumarate as the sole carbon source. Our results showed that *P. putida* is a promising microbial chassis for isoprenoids production with the improved capability of carbon utilization from lignocellulosic biomass for biofuel production.

## Materials and methods

### Strains and plasmid construction

All strains and plasmids used in this study are listed in Table 1. Strains and plasmids along with their associated information have been deposited in the public version of the JBEI Registry (https://public-registry.jbei.org; entries JPUB_019914 to JPUB_019988) and are available from the authors upon request. *P. putida* KT2440 was used for isoprenoid production, and *E. coli* DH5α was used for the general cloning.

**Table 1:** Strains and plasmids used in this study

Strains	Description	References
JPUB_019964 (Δ*phaABC*)	*P. putida* KT2440 deleted with the *phaA*-*phaB*-*phaC* gene cluster (PP_5003–PP_5005)	This study
JPUB_019965	*P. putida* KT2440 Δ*phaABC* ΔPP_2675	This study
JPUB_019966	*P. putida* KT2440 with pBbB1k-NudB	This study
JPUB_019967	*P. putida* KT2440 with pBbB5k-MTSA-T1-MK_sc_-PMK-PMD_sc_-NudB	This study
JPUB_019968	*P. putida* KT2440 with pBbB5k-AtoB-HMGS_sc_-HMGR_sc_-T1-MK_sc_-PMD_sc_	This study
JPUB_019969	*P. putida* KT2440 with pBbB5k-AtoB-HMGS_sa_-HMGR_sa_-T1-MK_sc_-PMD_sc_	This study
JPUB_019971	*P. putida* KT2440 with pBbB5k-MvaS_ef_-MvaE_ef_-T1-MK_sc_-PMD_sc_	This study
JPUB_019973	*P. putida* Δ*phaABC* with pBbB5k-AtoB-HMGS_sc_-HMGR_sc_-T1-MK_sc_-PMD_sc_	This study
JPUB_019974	*P. putida* Δ*phaABC* with pBbB5k-MvaS_ef_-MvaE_ef_-T1-MK_sc_-PMD_sc_	This study
JPUB_019975	*P. putida* Δ*phaABC* with pBbB5k-MvaS_ef_-MvaE_ef_-T1-MK_sc_-PMD_HKQ_	This study
JPUB_019976	*P. putida* Δ*phaABC* with pBbB5k-MvaS_ef_-MvaE_ef_-T1-MK_mm_-PMD_sc_	This study
JPUB_019977	*P. putida* Δ*phaABC* with pBbB5k-MvaS_ef_-MvaE_ef_-T1-MK_mm_-PMD_HKQ_	This study
JPUB_019978	*P. putida* Δ*phaABC* deleted with the *crc* gene (PP_5292)	This study
JPUB_019986	*P. putida* KT2440 with pBbB1k-EizS	This study
JPUB_019987	*P. putida* KT2440 with pBbB5k-MTSA-T1-MK_sc_-PMK-PMD_sc_-idi-ispA-T1-EizS	This study
JPUB_019988	*P. putida* Δ*phaABC* with pBbB5k-MTSA-T1-MK_sc_-PMK-PMD_sc_-idi-ispA-T1-EizS	This study

Plasmids	Description	Reference
JPUB_019914	pBbB1k-NudB	This study
JPUB_019916	pBbB5k-MTSA-T1-MK_sc_-PMK-PMD_sc_-NudB	This study
JPUB_019918	pBbB5k-AtoB-HMGS_sc_-HMGR_sc_-T1-MK_sc_-PMD_sc_	This study
JPUB_019970	pBbB5k-AtoB-HMGS_sa_-HMGR_sa_ -T1-MK_sc_-PMD_sc_	This study
JPUB_019920	pBbB5k-MvaS_ef_-MvaE_ef_-T1-MK_sc_-PMD_sc_	This study
JPUB_019922	pBbB5k-MvaS_ef_-MvaE_ef_-T1-MK_sc_-PMD_HKQ_	This study
JPUB_019923	pBbB5k-MvaS_ef_-MvaE_ef_-T1-MK_mm_-PMD_sc_	This study
JPUB_019925	pBbB5k-MvaS_ef_-MvaE_ef_-T1-MK_mm_-PMD_HKQ_	This study
JPUB_019933	pBbB1k-EizS	This study
JPUB_019935	pBbB5k-MTSA-T1-MK_sc_-PMK-PMD_sc_-idi-ispA-T1-EizS	This study
JPUB_019939	pK18-ppc	This study
JPUB_019941	pK18-pyc	This study
JPUB_019943	pK18-phaABC	This study
JPUB_019945	pK18-crc	This study
JPUB_018413	pNQ30-PP_2675	[28]
JPUB_019949	pBbB5k-MvaS_ef_-MvaE_ef_-T1-MK_mm_-PMD_HKQ_-crc	This study

Transformation of *P. putida* was performed by electroporation using a Bio-Rad (Bio-Rad Laboratories, Hercules, CA) MicroPulser preprogrammed EC3 setting (0.2 cm cuvettes with 50 μL cells, ~ 5 ms pulse, 3.0 kV) [29]. LB medium and LB agar medium were used for cell outgrowth and colony selection at 30 °C, respectively. Kanamycin (50 µg/mL) or gentamicin (30 µg/mL) was used as the selective antibiotics when needed. Gene knockout of *P. putida* was performed based on the homologous recombination followed by a suicide gene (*sacB*) counter-selection using modified pK18-mobSacB plasmids [30]. The genotypes of gene-knockout mutants were confirmed by colony PCR using specific primers, followed by DNA sequencing (GENEWIZ, South San Francisco, CA, USA).

### Isoprenol production in *P. putida*

An overview figure of typical process of isoprenol production and analysis is presented in the Supplementary information.

*P. putida* KT2440 strains bearing isoprenol pathway plasmids (Table 1) were used for isoprenol production. Starter cultures of all production strains were prepared by growing single colonies in LB medium containing 50 µg/mL kanamycin at 30 °C with 200-rpm shaking overnight. The starter cultures were diluted in 5 mL EZ-rich defined medium (Teknova, CA, USA) or M9 minimal medium [29], containing 10 g/L or 20 g/L glucose (1% or 2%, *w*/*v*), 25 µg/mL kanamycin in 50-mL culture tubes, and 0.5 mM IPTG was added to induce protein expression with OD_600_ at 0.4–0.6. When strains were cultivated in a 24-well microtiter plate, 2 mL medium was used and the plate was sealed with a gas-permeable film (Sigma-Aldrich, St. Louis, MO). When strains were cultivated in a 250-mL shake flask, 50-mL medium was used. l-glutamate was supplemented into the minimal medium at the indicated concentration when needed. For isoprenol production using *p*-coumarate as the carbon source, 10 g/L or 20 g/L (1% or 2%, *w*/*v*) *p*-coumarate was used to replace glucose in the EZ-rich defined medium. The *P. putida* cultures were incubated in rotary shakers (200 rpm) at 30 °C for 48 h.

### Evaluation of isoprenol consumption

*P. putida* strains (Table 1) were used to investigate isoprenol consumption. Starter cultures were prepared by inoculating glycerol stocks in LB medium at 30 °C with 200-rpm shaking overnight. The starter cultures were diluted with OD_600_ at 0.01 in 5 mL M9 minimal medium or EZ-rich defined medium (Teknova, CA, USA) containing 10 g/L glucose (1%, *w*/*v*) or no glucose (0%, *w*/*v*), added with 1 g/L isoprenol in 50-mL culture tubes. Amino acids (Additional file 1: Table S1) were added individually into the M9 minimal medium at desirable concentrations when needed. The *P. putida* cultures were incubated in rotary shakers (200 rpm) at 30 °C for 48 h. Blank media without strain inoculation were used in parallel to evaluate isoprenol evaporation loss.

### Quantification of isoprenol

The measurement and quantification of isoprenol were conducted by collecting 250 µL of cell culture and combining it with 250 µL of ethyl acetate containing 1-butanol (30 mg/L) as an internal standard. The mixture of ethyl acetate and cell culture was vigorously shaken for 15 min and subsequently centrifuged at 21,130 g for 3 min to separate the ethyl acetate phase from the aqueous phase. The ethyl acetate layer was collected and 1 µL was analyzed by gas chromatography-flame ionization detection (GC-FID, Thermo Focus GC) equipped with DB-WAX column (15 m, 0.32 mm inner diameter, 0.25 µm film thickness, Agilent, USA). The GC oven was programmed as follows: 40–100 °C at 15 °C/min, 100–230 °C at 40 °C/min, held at 230 °C for 2 min. The inlet temperature was 200 °C.

### Production and quantification of epi-isozizaene

*P. putida* KT2440 bearing the pathway plasmid (Table 1) was used for epi-isozizaene production. Starter cultures of all production strains were prepared by growing single colonies in LB medium containing 50 µg/mL kanamycin at 30 °C with 200-rpm shaking overnight. The starter cultures were diluted in a 5 mL EZ-rich defined medium (Teknova, CA, USA) containing 10 g/L glucose (1%, *w*/*v*), 25 µg/mL kanamycin in 50-mL culture tubes. 0.5 mM IPTG was added to induce protein expression with OD_600_ at 0.4–0.6, and 0.5 mL nonane (10%, v/v) was added as a solvent overlay. The *P. putida* cultures were incubated in rotary shakers (200 rpm) at 30 °C for 72 h.

For epi-isozizaene measurement, the solvent overlay was sampled and centrifuged at 21,130 g for 3 min. The overlay layer was collected and diluted with ethyl acetate containing 5 mg/L guaiazulene as the internal standard. 1 µL was analyzed by Agilent GC–MS equipped with HP-5 column (Agilent, USA). The GC oven was programmed from 40 °C (held for 3 min) to 295 °C at 15 °C/min. The concentration of epi-isozizaene was estimated using the TIC areas with alternative standard (−)-trans-caryophyllene as described in a previous study [31].

### Quantification of metabolites

The concentrations of glucose and organic acids from the culture were measured with an Agilent 1100 Series HPLC system, equipped with an Agilent 1200 Series refractive index detector (RID) (Agilent Technologies, CA) and Aminex HPX-87H ion-exclusion column as described in a previous study [32]. The quantification of glucose and organic acids was calibrated with authentic standards.

For metabolomics analysis, 1.5 mL cell culture was collected at 24 and 48 h and centrifuged at 13,000 g for 1 min at room temperature. The cell pellet was quenched with 250 µL methanol, vortexed, and stored at − 20 °C. For sample preparation, 250 µL water was added to the methanol lysate and mix thoroughly. Centrifuge the methanol/water lysate at 13,000 g for 10 min at 4 °C. The supernatant was filtered by a Millipore Amicon Ultra 3 kDa cut-off filter (Billerica, MA) at 13,000 g at − 2 °C for 30–60 min until most of the sample has been filtered. The intracellular metabolite concentrations were quantified by liquid chromatography and mass spectrometry (LC–MS) methods as previously described by Baidoo et al. (with reference to note 6) [33].

## Results

### Engineering *P. putida* for isoprenol production

**Fig. 2:**
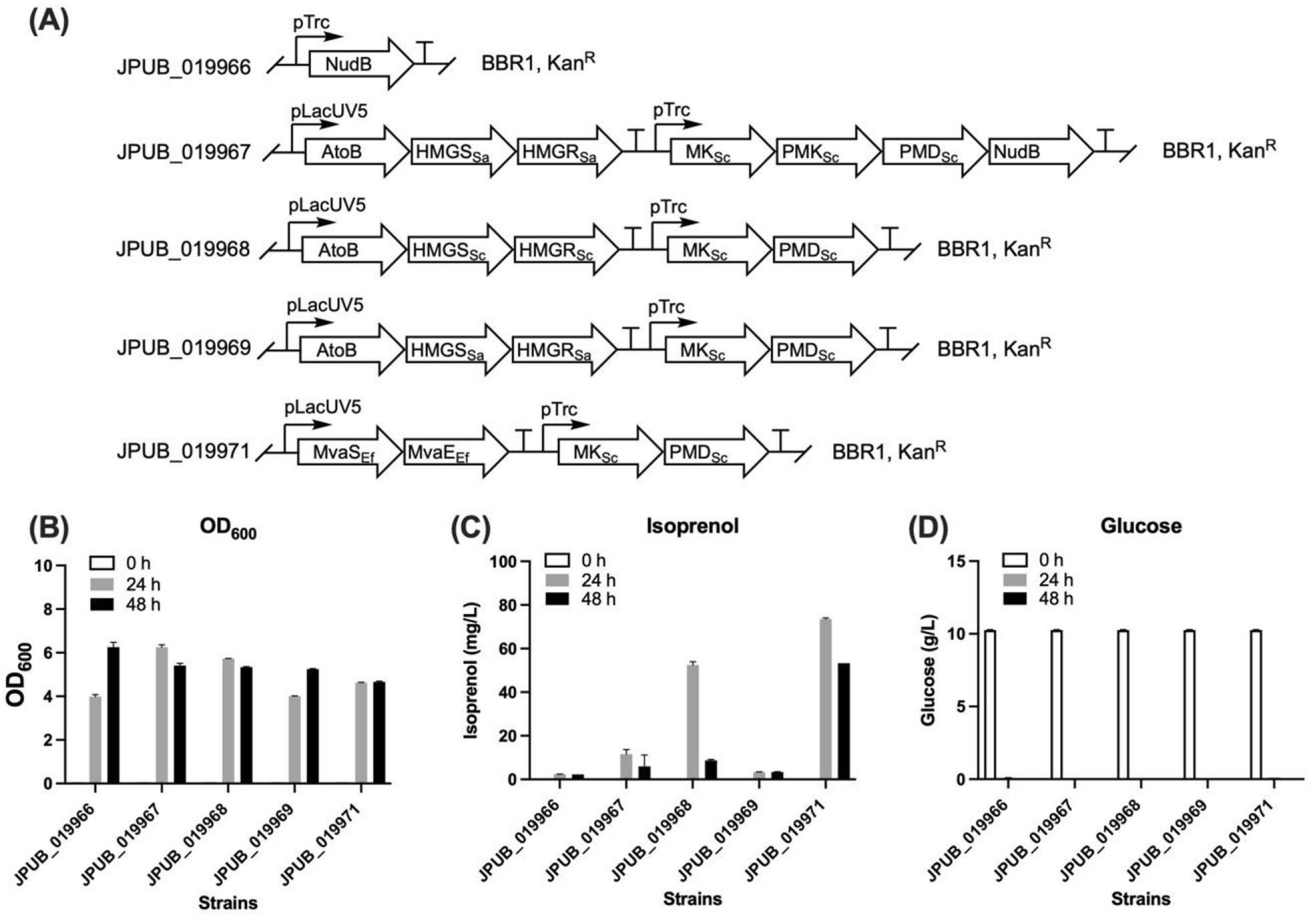
Engineering heterologous pathway for isoprenol production in *P. putida*. **A** Configuration of *P. putida* strains with the engineered isoprenol pathway plasmids. **B**–**D** Production results by the engineered *P. putida* strains from 1% glucose. **B** Cell growth. The initial OD_600_ of 0 h was set at 0.05. **C** Isoprenol production. Isoprenol levels of different strains were not detectable at 0 h; **D** Glucose consumption. Error bars indicate one standard deviation of triplicates. NudB, dihydroneopterin triphosphate diphosphatase (*E. coli*); AtoB, acetoacetyl-CoA synthase (*E. coli*); HMGS, HMG-CoA synthase; HMGR, HMG-CoA reductase; MvaS, HMG-CoA synthase; MvaE, HMG-CoA reductase; MK, mevalonate kinase; PMK, phosphomevalonate kinase; PMD, phosphomevalonate decarboxylase. The footnote of enzymes indicates their sources: Sc, *S. cerevisiae*; Sa, *Staphylococcus aureus*; Ef, *Enterococcus faecalis*. BBR1, broad host range replication origin; Kan^R^, kanamycin-resistant antibiotic marker

*P. putida* natively possesses the MEP pathway for isoprenoids biosynthesis. To produce isoprenol in *P. putida*, we first attempted to use the endogenous MEP pathway and overexpressed the *E. coli* dihydroneopterin triphosphate diphosphatase (NudB) that has a promiscuous activity to catalyze the conversion of IPP to IP which is hydrolyzed to isoprenol by endogenous phosphatases [22]. In this case, *P. putida* KT2440 was transformed with a high-copy plasmid pBbB1k-NudB (Table 1) using a modified broad host range replication origin BBR1 [34] and a Trc promoter which works both in *E. coli* and *P. putida*. The engineered *P. putida* strain (JPUB_019966, Table 1, Fig. 2A) could produce a low level of isoprenol at 2 mg/L after 48 h from 1% glucose (Fig. 2C).

We then engineered a heterologous MVA pathway, which has shown high isoprenol production in *E. coli* [14]. To construct the MVA pathway, two operons were used to express the MVA pathway genes onto the plasmid backbone of pBbB5k. The expression of the top portion of the MVA pathway (AtoB, HMGS, HMGR) was driven by a LacUV5 promoter, and the expression of the bottom portion enzymes (MK, PMK, PMD) as well as NudB were driven by a Trc promoter. The resulting engineered *P. putida* strain (JPUB_019967, Table 1, Fig. 2A) produced up to 12 mg/L isoprenol at 24 h and the titer decreased at a later time point (Fig. 2C).

Finally, we engineered the IPP-bypass MVA pathway to compare the isoprenol production by using the promiscuous activity of PMD [22] in *P. putida*. Three different MVA pathway top-portion operons (MevT, MTSA, and MvaES) were studied, which the HMGS and HMGR genes are from *Saccharomyces cerevisiae*, *Staphylococcus aureus*, and *Enterococcus faecalis*, respectively (JPUB_019968 to JPUB_019971, Table 1, Fig. 2A，Additional file 1: Figure S9). Results showed that the highest isoprenol production (up to 74 mg/L after 24 h) was observed in the strain with the MvaES top portion operon (Fig. 2C). Compared with the strain using the original MVA pathway, strains with the IPP-bypass MVA pathways (except the one using the MTSA top portion operon) showed a 4- to 6-fold increase of isoprenol production. This suggests the IPP-bypass MVA pathway can be used in *P. putida* for isoprenol production and it shows higher efficiency than the original MVA pathway or the endogenous MEP pathway.

### Optimization of isoprenol production in *P. putida*

Given that *E. coli* has shown much higher isoprenol production [24] than what we achieved in *P. putida*, we compared the metabolic difference between *P. putida* and *E. coli* to identify limiting steps and target them to optimize isoprenol production in *P. putida.* We used the published ^13^C-metabolic flux data of *P. putida* [11] and *E. coli* [35] for the comparison (Additional file 1: Figure S1). Interestingly, we found *P. putida* derived threefold more carbon flux from acetyl-CoA to TCA cycle compared with *E. coli*, which indicated less acetyl-CoA availability in *P. putida* for isoprenol production. Another difference is *P. putida* possesses both phosphoenolpyruvate carboxylase (*ppc*) and pyruvate carboxylase (*pyc*) that can direct 2.8-fold more flux from glycolysis to the TCA cycle, whereas the *pyc* gene does not exist in *E. coli*. In addition, *P. putida* can naturally synthesize polyhydroxyalkanoate (PHA) from acetyl-CoA as a carbon sink [36]. Therefore, we attempted to knockout *ppc*, *pycAB*, and *phaABC* (PHA synthase) genes to improve acetyl-CoA pool and isoprenol production in *P. putida*. As the Additional file 1: Figure S2 shows, the knockout of *ppc* and *pycAB* genes did not improve isoprenol production compared with the wild-type strain. The double knockout of *ppc* and *pycAB* genes produced an even lower amount of isoprenol. However, the deletion of *phaABC* genes increased 24% of isoprenol production compared with the wild-type strain during the screening in a microtiter plate, which suggests they are promising targets.

**Fig. 3:**
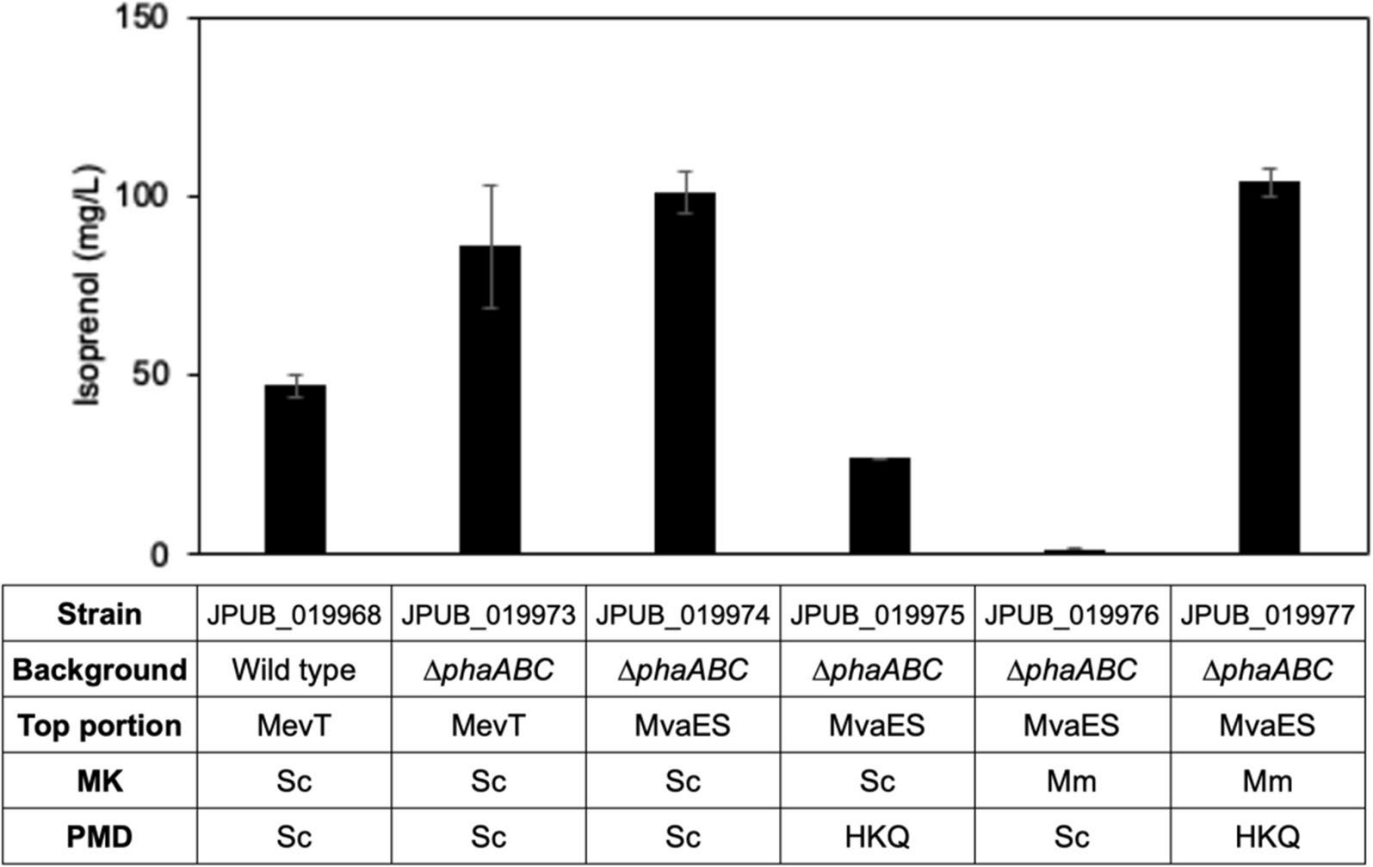
Optimizing isoprenol production. Isoprenol production from 2% glucose with different background strains, top-portion MVA pathway, and MK-PMD genes. Error bars indicate one standard deviation of triplicates. MK, mevalonate kinase; PMD, phosphomevalonate decarboxylase. Sc, *S. cerevisiae*; Mm, *Methanosarcina mazei*; HKQ, a mutant of PMD_Sc_ containing three mutations (R74H, R147K, and M212Q)

On the other hand, we noticed that the production results in the previous section showed decreased isoprenol levels and depleted glucose after 24 h (Fig. 2C). This indicated 1% glucose concentration might be insufficient to support a 48-h production process. Thus, we increased the initial glucose concentration to 2%, and the IPP-bypass MVA pathway with the MevT operon improved the isoprenol production level from 9 mg/L to 47 mg/L after 48 h (Fig. 3). When using the Δ*phaABC* strain, isoprenol production was further increased to 86 mg/L from 2% glucose at 48 h (Fig. 3). By applying this new condition (Δ*phaABC* strain + 2% glucose) to the best producing pathway (IPP-bypass_MvaES), the engineered *P. putida* strain (JPUB_019974, Table 1) reached 101 mg/L isoprenol production after 48 h (Fig. 3), which was a ~ 2-fold improvement of isoprenol production from the starting conditions (53 mg/L from the wild-type strain and 1% glucose).

Given that MK and PMD are key steps to converting MVA to isoprenol, we also tested different combinations of the MK-PMD gene cassettes to optimize isoprenol production. Based on previous results in *E. coli* [24], we selected two efficient enzyme versions, MK_Mm_ (MK from *Methanosarcina mazei*) and PMD_HKQ_ (a mutant of PMD_Sc_ containing three mutations [37]) to construct four combinations of the MK-PMD cassette. Results showed that the strain with MK_Mm_-PMD_HKQ_ (JPUB_019977, Table 1) produced the highest isoprenol at 104 mg/L after 48 h from 2% glucose in a culture tube (Fig. 3). More production details were studied by culturing this best producer in a shake flask. As the Additional file 1: Figure S3 shows, glucose was not fully depleted and ~ 2.5 g/L of residual glucose was detected in the culture after 48 h, which suggested the initial glucose concentration at 2% was sufficient in supporting a 48-h production. No significant amounts of organic acids were detected as fermentative by-products except the small amount of acetate (0.8 g/L) and succinate (0.2 g/L) observed only at 24 h. While the isoprenol titer was lower in the shake flask (80 mg/L), it might be attributed to potentially faster isoprenol evaporation in the flask than in the culture tube. Unlike the top-portion MVA pathway, changing MK-PMD genes did not significantly improve isoprenol production. Collectively, we engineered the IPP-bypass MVA pathway in *P. putida* KT2440 for isoprenol production and achieved the highest production titer from glucose at up to 104 mg/L (c.f. the maximum theoretical yield from glucose is 0.319 g/g glucose [38]).

### Investigation of isoprenol consumption in *P. putida*

**Fig. 4:**
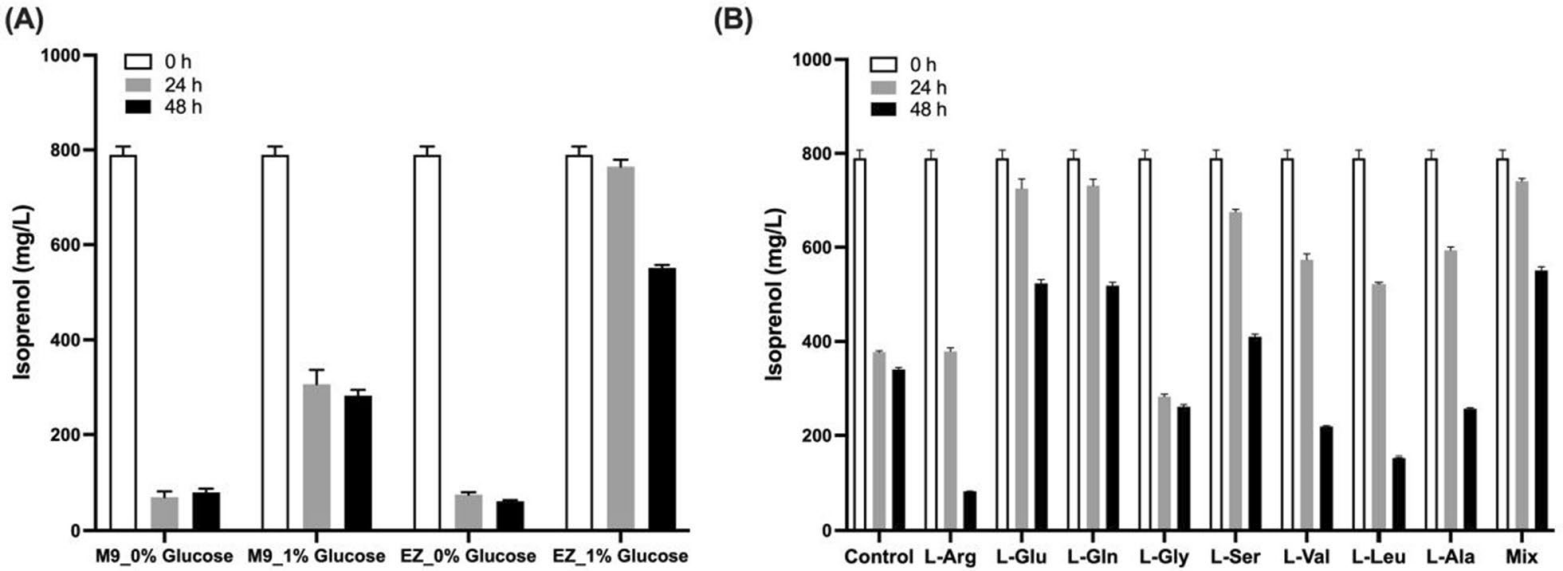
Investigation of isoprenol consumption in *P. putida*. **A** Isoprenol consumption by *P. putida* Δ*phaABC* strain (JPUB_019964) in M9 minimal medium and EZ-rich medium containing 1% glucose or no glucose, respectively. **B** Isoprenol consumption by *P. putida* Δ*phaABC* strain in M9 minimal medium supplemented with individual amino acid. The working concentrations of 8 amino acids were shown in Additional file 1: Table S1. Control, no amino acid supplemented; Mix, the mixture of all 8 amino acids with the same individual concentration. Error bars indicate one standard deviation of triplicates

While the above isoprenol production was performed in the EZ-rich defined medium, it is also important to perform the production in the minimal medium, which is more frequently used for bioreactor fermentation and metabolic flux analysis [24]. Using the highest isoprenol producer (JPUB_019977, Table 1) from the EZ-rich defined medium, we tested isoprenol production in M9 minimal medium but observed low levels of isoprenol (~ 1 mg/L) after 48 h from 2% glucose (Additional file 1: Figure S4). Since *P. putida* has shown the capability of utilizing isoprenol as a carbon source [28], this urged us to investigate the difference between the two media that were used for isoprenol production. We first compared isoprenol consumption in the M9 minimal medium and EZ-rich medium. It was observed that the addition of glucose could help to preserve isoprenol in the medium, and the consumption was significantly slower in the EZ-rich medium when glucose is present (5 mg/L/hour) than in the M9 minimal medium (11 mg/L/hour) in 48 h (Fig. 4A).

To find out which other component of the EZ-rich medium contributed to slowing down the isoprenol consumption, we compared the recipes of two media and identified 8 amino acids that are present at a higher concentration in the EZ-rich medium formulation (Additional file 1: Table S1). By supplementing these 8 amino acids individually into the M9 minimal medium at the same concentration used in the EZ-rich medium, surprisingly, we found that the addition of L-glutamate (L-Glu) or L-glutamine (L-Gln) preserved isoprenol to a similar level that was observed in the EZ-rich medium (Fig. 4B). We chose L-Glu as the supplement to investigate isoprenol production in the minimal medium and observed that the addition of 6 mM L-Glu resulted the highest isoprenol production level to 15 mg/L after 48 h (Additional file 1: Figure S4), which is nearly a 15-fold increase compared with the previous level without any supplements (~ 1 mg/L).

**Fig. 5:**
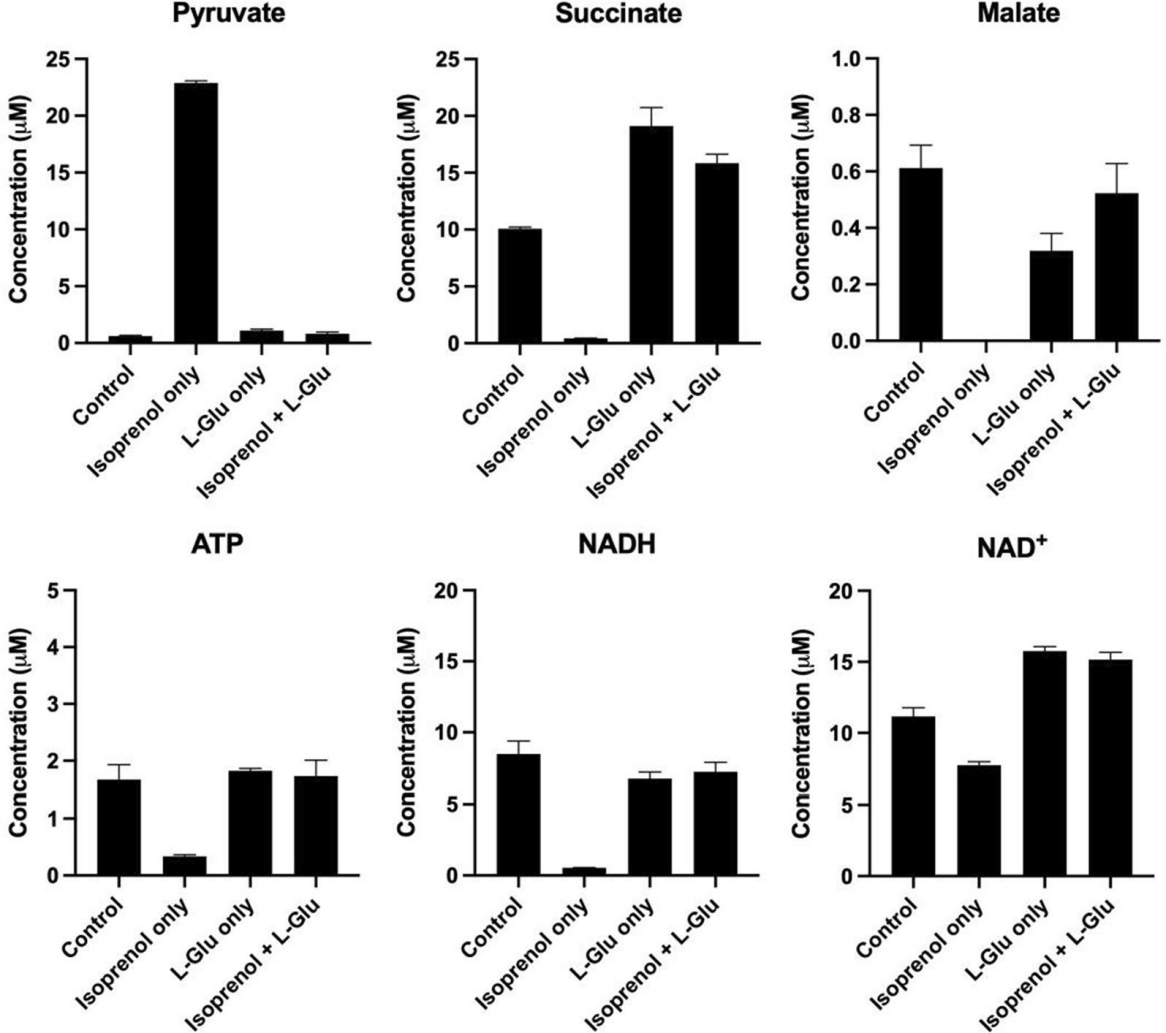
Metabolite analysis of l-glutamate supplement during isoprenol consumption in *P. putida* (JPUB_019964). Metabolites under four conditions were investigated after 24 h. Control, no isoprenol and no l-glutamate added; Isoprenol only, 1 g/L isoprenol added; l-Glu only, 6 mM l-glutamate added; Isoprenol + l-Glu, 1 g/L isoprenol and 6 mM l-glutamate added. Error bars indicate one standard deviation of triplicates

Based on the findings of the L-Glu supplementation experiment, we continued to investigate the mechanism that L-Glu involves in isoprenol preservation in *P. putida*. We compared the intracellular metabolites between the conditions with and without the L-Glu supplement. When isoprenol is presented in the medium without the L-Glu supplement, it showed a significant difference in metabolites of central carbon and energy metabolism after 24 h (Fig. 5). Although isoprenol could provide an additional carbon source, the difference in pyruvate, succinate, malate, ATP, NADH, and NAD^+^ levels indicated an insufficient energy supply and imbalanced redox compared with the control group. In contrast, supplementing L-Glu restored those metabolites to comparable levels to the control group (Fig. 5). Since L-Glu is considered as a favored carbon source for *P. putida* [39], the L-Glu-mediated prevention of isoprenol self-consumption could be attributed to carbon catabolite repression (CCR). To verify this, we deleted the CCR regulator gene (*crc*) from the *P. putida* chromosome and studied the isoprenol consumption with the Δ*crc* strain (JPUB_019978, Table 1). Results showed the prevention of isoprenol from self-consumption by supplementing L-Glu was significantly reduced when *crc* is deleted (222 mg/L, Additional file 1: Figure S5B), compared with the strain without *crc* deletion (523 mg/L, Fig. 4B). This suggests L-Glu assisted isoprenol preservation in *P. putida* may be attributed to CCR, in which L-Glu is a preferred carbon source, rather than isoprenol, in supporting rapid cell growth [40]. However, overexpressing *crc* with the isoprenol pathway did not increase isoprenol production, but even lowered the production titer (6 mg/L, Additional file 1: Figure S6).

### Isoprenol production using *p*-coumarate as a carbon source

**Fig. 6:**
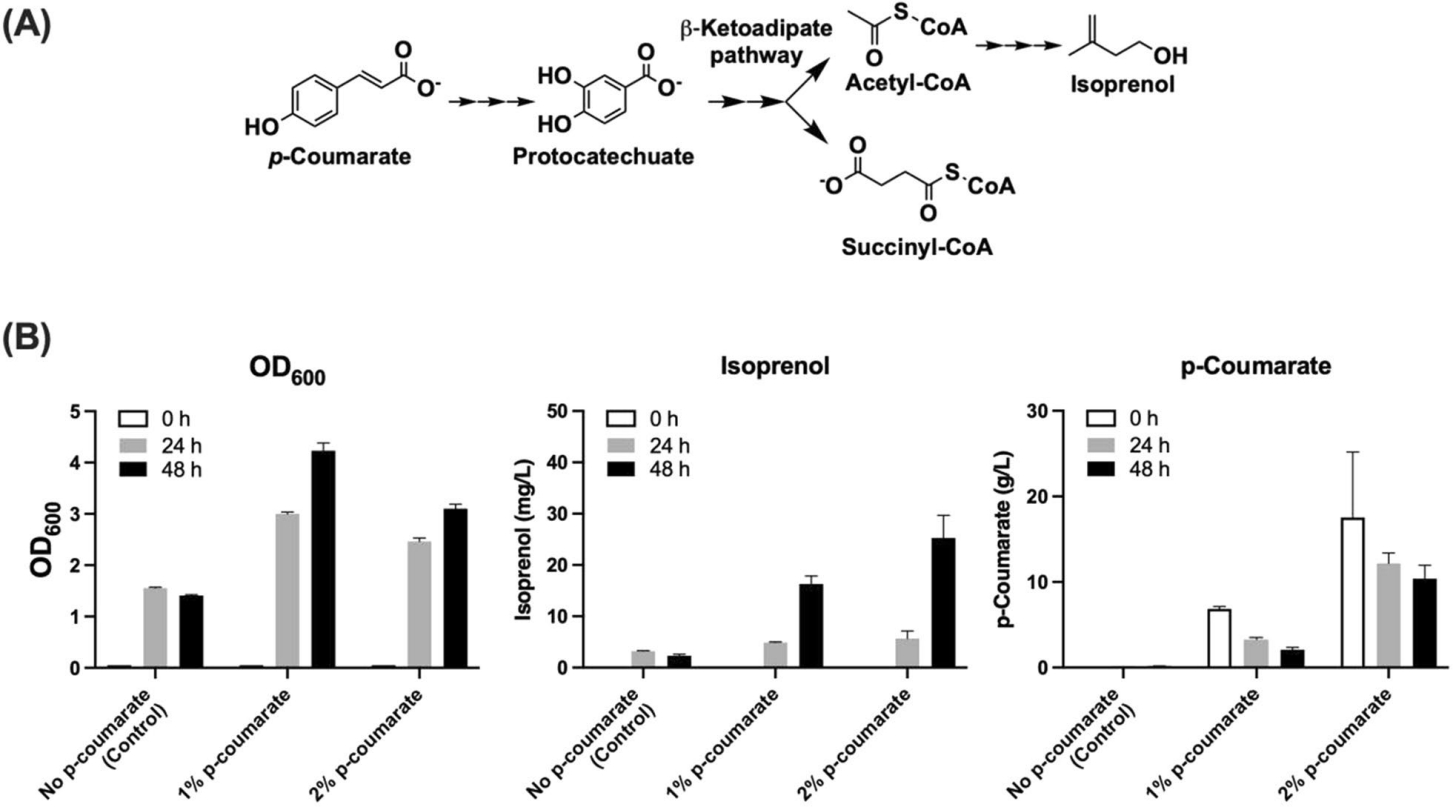
Isoprenol production from *p*-coumarate in the engineered *P. putida*. **A** *p*-Coumarate catabolic pathway; **B** Isoprenol production by engineered *P. putida* (JPUB_019977). EZ-rich medium was used as the base medium by changing the carbon source to 1% or 2% *p*-coumarate. The blank EZ-rich medium without any carbon sources was used as a control. Error bars indicate one standard deviation of triplicates. The initial OD_600_ of 0 h was set at 0.05. Isoprenol levels were not detectable at 0 h

*P*-Coumarate is a prominent compound used as a representative lignin-derived aromatics and there are efforts to increase *p*-coumarate content in lignocellulosic biomass [41]. We attempted to use *p*-coumarate as the carbon source to investigate isoprenol production in the engineered *P. putida* strain. Results showed that the engineered *P. putida* strain (JPUB_019977, Table 1) can produce up to 25 mg/L isoprenol from 2% *p*-coumarate (c.f. the maximum theoretical yield from *p-coumarate* is 0.273 g/g *p*-coumarate) after 48 h (Fig. 6), which is 24% of the isoprenol titer achieved from 2% glucose. We observed that the cell growth at 2% *p*-coumarate was 27% lower than the 1% *p*-coumarate condition after 48 h. More residual *p*-coumarate was detected in the medium for the 2% condition (Fig. 6), suggesting a higher concentration of *p*-coumarate may inhibit cell growth. Though the isoprenol titer was lower from *p*-coumarate than from glucose, it showed the possibility of utilizing aromatics as well as sugars as the carbon source in biofuel production. This demonstrates that *P. putida* is a promising host for the comprehensive conversion of carbons from lignocellulosic biomass for bio-based production.

### Engineering *P. putida* for other larger terpenes production

**Fig. 7:**
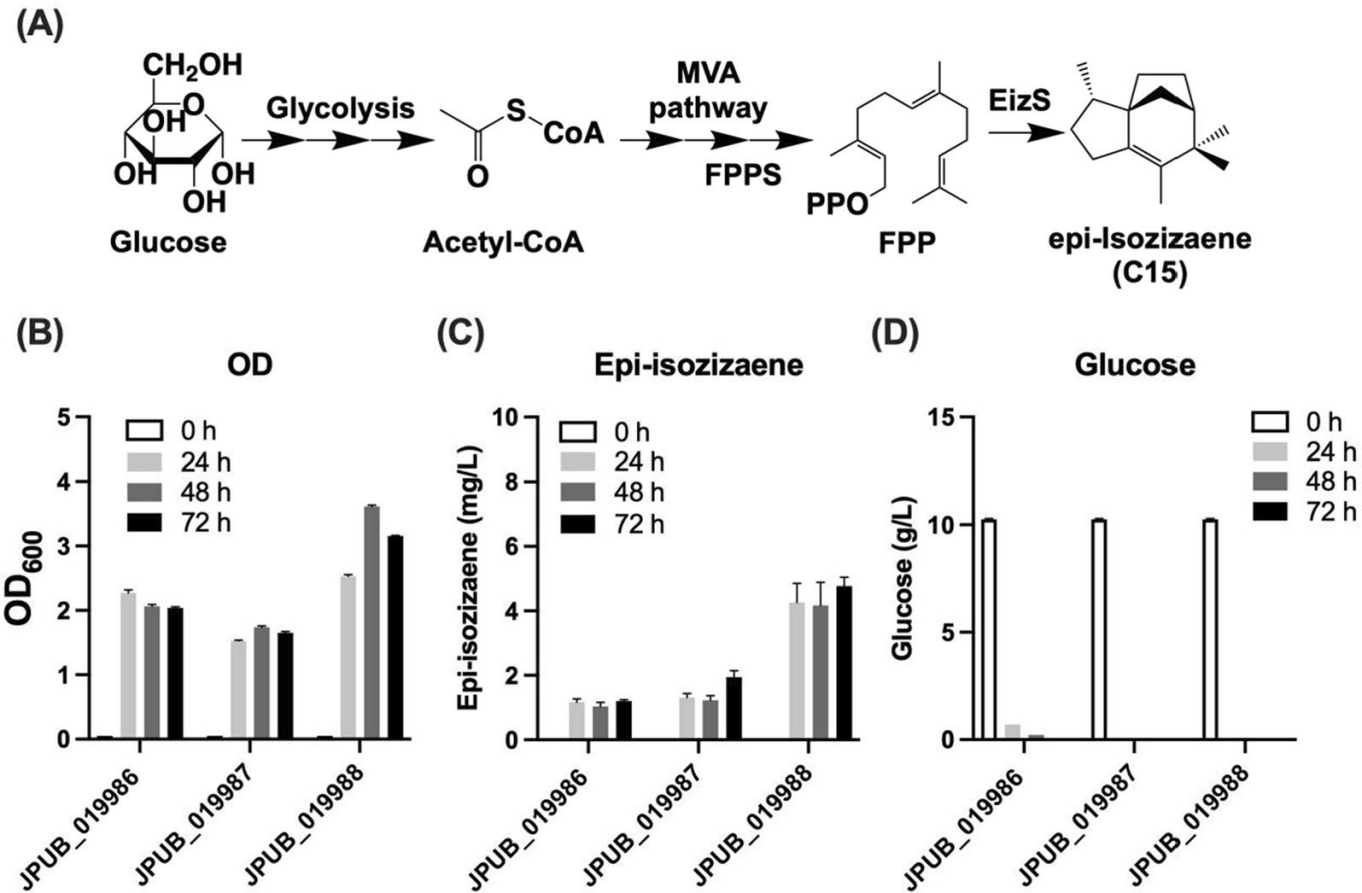
Production of sesquiterpene in the engineered *P. putida*. **A** Epi-isozizaene synthesis pathway from glucose. **B**–**D** Production results by the engineered *P. putida* strains from 1% glucose. **B** Cell growth; **C** Epi-isozizaene production; **D** Glucose consumption. Error bars indicate one standard deviation of triplicates. FPP, farnesyl diphosphate; FPPS, farnesyl diphosphate synthase; EizS, epi-isozizaene synthase from *Streptomyces coelicolor*. Error bars indicate one standard deviation of triplicates

To expand the isoprenoid production profile in *P. putida* via the MVA pathway, we engineered the MVA pathway for monoterpenes and sesquiterpenes. We chose two monoterpenes (limonene, C10: 49 mg/L, 1,8-cineole, C10: 6 mg/L; Additional file 1: Figure S8) and one sesquiterpene (epi-isozizaene, C15) as targets for production. To demonstrate the capability of producing the larger terpene molecule (C15), we performed a similar strategy in engineering epi-isozizaene production. Herein, the MEP pathway was used as a control by overexpressing the epi-isozizaene synthase. As shown in Fig. 7, the MVA pathway (2 mg/L) showed a higher level of epi-isozizaene than the MEP pathway (1 mg/L). By applying the Δ*phaABC* strain for the MVA pathway, a higher production for epi-isozizaene (5 mg/L) was observed from 1% glucose, which is consistent with the results obtained from isoprenol production. However, compared with isoprenol, the production level of epi-isozizaene was much lower, and more efforts are needed to optimize the pathway for sesquiterpenes.

## Discussion

In this study, we engineered the heterologous MVA pathway in *P. putida* KT2440 to produce isoprenoids, including isoprenol (C_5_) and epi-isozizaene (C_15_). Unlike the *E. coli* system, the use of a heterologous MVA pathway showed very limited improvement of isoprenoid production (Figs. 2, 7). These results are consistent with a previous report [26] and the reason might be the result of the distinct central metabolism in *P. putida* and its different flux distribution with acetyl-CoA (Additional file 1: Figure S1). For isoprenol, we also engineered the IPP-bypass MVA pathway, and it showed advantages compared with using the MEP and the original MVA pathway. The highest isoprenol titer from engineered *P. putida* was 104 mg/L from 2% glucose.

While the use of the IPP-bypass MVA pathway made a substantial improvement during isoprenol production, this is still much lower than the batch culture titer (~ 2,500 mg/L) reported in *E. coli* [24]. Compared with the *E. coli* system, the low isoprenol titer might be attributed to two reasons. First, the isoprenol degradation pathway in *P. putida* competes with the synthesis pathway, leading to a reduced accumulation of isoprenol. In contrast, *E. coli* does not show the capability of consuming isoprenol as a carbon source. Due to isoprenol consumption being very significant in *P. putida* (up to 714 mg/L isoprenol was consumed in 24 h, Fig. 4A), this could be one of the main reasons that the engineered *P. putida* cannot show a comparable isoprenol titer to the similarly engineered *E. coli* strains. We tried deleting a gene (PP_2675) reported to be associated with *P. putida*’s growth on isoprenol [28], but this deletion did not improve the isoprenol titer (Additional file 1: Figure S7). As many genes have been identified as being involved in isoprenol catabolism [28], additional gene deletion may be required to achieve reduction in isoprenol catabolism and degradation without compromising isoprenol production. Second, the balancing of isoprenol-pathway enzymes was harder to achieve in *P. putida* than in *E. coli* as the number and the variety of plasmids are limited in *P. putida*. In the *E. coli* system, two plasmids could be used for isoprenol production to achieve a well-balanced pathway proteins expression for the best isoprenol production. For example, the 1st plasmid was selected to be a medium-to-low copy plasmid to drive the top MVA portion genes (*atoB*, *HMGS*, *HMGR*) to reduce accumulation of the final metabolite (mevalonate) and prevent the substrate inhibition against the next enzyme (MK) in the pathway. The 2nd plasmid contained a high copy origin and drove *MK* and *PMD* genes expression under a strong promoter as higher level expression of these enzymes is required to drive the pathway toward the product [24]. Therefore, to optimize the balancing of pathway enzymes in *P. putida*, a systematic comparison of pathway constructs such as using a 1-plasmid vs 2-plasmid system, varying plasmid copy numbers, and changing the strength of the promoter or RBS in driving different pathway genes will be required as previously shown in the *E. coli* isoprenoid studies [42].

As *P. putida* consumes isoprenol, we investigated the possibilities of preventing the consumption of isoprenol by supplementing specific medium components. Interestingly, we found supplementing l-Glu in the culture medium showed a significant preservation of isoprenol. Using metabolomics, we revealed the difference of intracellular metabolites and attempted to explain the possible scenarios during isoprenol degradation. The metabolites analysis showed an insufficient energy availability and an imbalanced redox status during isoprenol degradation. This may be associated with the alcohol degradation mechanism as *P. putida* utilizes pyrroloquinoline quinone (PQQ)-dependent alcohol dehydrogenases for alcohol degradation [43], which may change the balance of cellular redox when processing isoprenol degradation. In addition, since l-Glu is a precursor of PQQ biosynthesis [44], supplementing l-Glu could increase substrate availability toward PQQ biosynthesis, which might contribute to the rebalancing of redox status as well as restoring the cellular metabolism. On the other hand, a few studies reported the development of isoprenol utilization pathways for isoprenoid synthesis, such as isopentenol utilization pathway (IUP) [45] and isoprenoid alcohol (IPA) pathway [46]. In these pathways, the alcohol kinase (e.g., yeast choline kinase, [45]) was identified and used for isoprenol phosphorylation, which indicated alcohol phosphorylation might be an alternative route besides alcohol dehydrogenation related to isoprenol degradation.

As *P. putida* is an emerging microbial host, there are still many challenges to engineer this host as a bioproduction workhorse. For example, even though some *P. putida* species can utilize xylose as a carbon source, the most widely studied *P. putida* microbial platform (KT2440) cannot naturally utilize xylose. Thus, engineering for the simultaneous utilization of glucose, xylose, and lignin-derived aromatic substrates may need additional efforts to achieve optimal carbon utilization without comprising the production yields [32, 47]. The versatile metabolism of *P. putida* which allows it to survive with broad substrates also brings issues of the self-degradation of biosynthetic products. These issues could be challenging to overcome since multiple genes and regulations may be involved in the degradation process [28]. Additionally, the polyploid property nature of *P. putida* may increase the instability of using a high-copy plasmid for gene expression [48], and consistent with this we observed significant variations among colonies when screening for productions. Even with these issues, the unique capability of *P. putida* to utilize lignin-derived intermediates and aromatics as carbon sources are clear advantages over the widely used microbial hosts such as *E. coli* and *S. cerevisiae* as a next-generation industrial microbial host for converting lignocellulosic biomass to biofuels and bioproducts. In this study, we demonstrated that the engineered *P. putida* strains can utilize *p*-coumarate, as the sole carbon source to produce isoprenol. It is foreseeable that *P. putida* can achieve an economically feasible production of isoprenol and other bio-based products from lignocellulosic biomass via systematic strain engineering combining the efforts of computation and analytics using the Design-Build-Test-Learn research cycle [49].

## Conclusions

*P. putida* can naturally utilize broad carbon sources and is tolerant to xenobiotics, which shows great potential to be developed as an emerging industrial microbial workhorse especially in maximally converting carbon from lignocellulosic biomass to biofuels and bioproducts. In this study, we engineered the heterologous MVA pathway in *P. putida* KT2440 to produce isoprenoids, including isoprenol (C_5_) and epi-isozizaene (C_15_). IPP-bypass MVA pathway showed advantages during isoprenol production. Through comparing flux distribution and identifying gene-knockout target, we optimized the production strain to achieve an increase of isoprenol production to 104 mg/L in a batch flask experiment. Due to the isoprenol degradation in *P. putida*, we investigated the strategy to prevent self-consumption of isoprenol, and supplementation of l-Glu in the medium was found to show significant preservation for isoprenol. The engineered *P. putida* strain can also produce isoprenol using *p*-coumarate as the sole carbon source. Our results presented a good demonstration of developing *P. putida* as a new microbial chassis for biofuel production with improved carbon utilization from lignocellulosic biomass.

## Supplementary Information

**Additional file 1: Table S1.** Identified 8 amino acids from the EZ-rich medium and their working concentrations. **Table S2.** Strains and plasmids used in monoterpene production. **Figure S1.** Comparison of carbon flux distribution between *E. coli* and *P. putida*. **Figure S2.** Comparison of isoprenol production with *P. putida* gene-knockout strains. **Figure S3.** Isoprenol production with *P. putida* Δ*phaABC* strain. **Figure S4.** Isoprenol production in M9 minimal medium supplemented with L-Glu. **Figure S5.** Investigation of isoprenol consumption for *P. putida *Δ*crc *strain. **Figure S6.** Isoprenol production with *crc* overexpression by *P. putida *Δ*phaABC *strain. **Figure S7.** Isoprenol consumption and production in *P. putida *Δ*phaABC* ΔPP_2675 strain. **Figure S8.** Production of monoterpene in the engineered *P. putida*. **Figure S9.** Targeted proteomics of IPP-bypass MVA pathway in isoprenol production.

## Data Availability

The dataset supporting the conclusions of this article is available in the JBEI’s Experiment Data Depot (https://edd.jbei.org/) and the strain information is available in the public version of the JBEI Registry (https://public-registry.jbei.org).
