# Supplementary material for: Engineering isoprenoids production in metabolically versatile microbial host Pseudomonas putida
Source: Biotechnol Biofuels Bioprod. 2022 Dec 12;15:137. doi: 10.1186/s13068-022-02235-6 (PMC9743605; doi:10.1186/s13068-022-02235-6)
Supplement: Supplementary file 1 — Additional file 1: Table S1. Identified 8 amino acids from the EZ-rich medium and their working concentrations. Table S2. Strains and plasmids used in monoterpene production. Figure S1. Comparison of carbon flux distribution between E. coli and P. putida. Figure S2. Comparison of isoprenol production with P. putida gene-knockout strains. Figure S3. Isoprenol production with P. putida ΔphaABC strain. Figure S4. Isoprenol production in M9 minimal medium supplemented with L-Glu. Figure S5. Investigation of isoprenol consumption for P. putida Δcrc strain. Figure S6. Isoprenol production with crc overexpression by P. putida ΔphaABC strain. Figure S7. Isoprenol consumption and production in P. putida ΔphaABC ΔPP_2675 strain. Figure S8. Production of monoterpene in the engineered P. putida. Figure S9. Targeted proteomics of IPP-bypass MVA pathway in isoprenol production. [file 13068_2022_2235_MOESM1_ESM.docx]

Additional file

Engineering isoprenoids production in metabolically versatile microbial host *Pseudomonas putida*

Xi Wang^1,2^, Edward E. K. Baidoo^1,2^, Ramu Kakumanu^1,2^, Silvia Xie^1,2,3^, Aindrila Mukhopadhyay^1,2^, Taek Soon Lee^1,2,*^

^1^ Joint BioEnergy Institute (JBEI), 5885 Hollis St., Emeryville, CA 94608, USA

^2^ Biological Systems & Engineering Division, Lawrence Berkeley National Laboratory, Berkeley, CA 94720, USA

^3^ Department of Molecular & Cell Biology, University of California, Berkeley, CA 94720, USA

^*^ Corresponding author, Joint BioEnergy Institute, 5885 Hollis Street, Emeryville, CA 94608, USA.

E-mail address: [tslee@lbl.gov](mailto:tslee@lbl.gov) (T. S. Lee).

**Overview Figure of Tube Culturing Experiment**


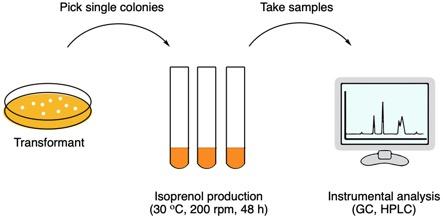


**Additional Methods**

**Monoterpene production and quantification in *P. putida***

*P. putida* KT2440 strains bearing monoterpene pathway plasmids (Table S2) were used for limonene or 1,8-cineole production. Starter cultures of all production strains were prepared by growing single colonies in LB medium containing 50 µg/mL kanamycin at 30°C with 200-rpm shaking overnight. The starter cultures were diluted in 5 mL EZ-rich defined medium (Teknova, CA, USA), containing 10 g/L glucose (1%, w/v), 25 µg/mL kanamycin in 50-mL culture tubes, and 0.5 mM IPTG was added to induce protein expression with OD_600_ at 0.4 – 0.6. 0.5 mL dodecane (10%, v/v) was added as a solvent overlay. The *P. putida* cultures were incubated in rotary shakers (200 rpm) at 30°C for 48 hours. The measurement and quantification of limonene and 1,8-cineole were conducted as described previously [1].

**Table S1** Identified 8 amino acids from the EZ-rich medium and their working concentrations

| **No.** | **Amino acid** | **Concentration** |
| --- | --- | --- |
| 1 | L-Arginine HCl (L-Arg) | 5.2 mM |
| 2 | L-Glutamic acid, potassium salt (L-Glu) | 0.6 mM |
| 3 | L-Glutamine (L-Gln) | 0.6 mM |
| 4 | L-Glycine (L-Gly) | 0.8 mM |
| 5 | L-Serine (L-Ser) | 10 mM |
| 6 | L-Valine (L-Val) | 0.6 mM |
| 7 | L-Leucine (L-Leu) | 0.8 mM |
| 8 | L-Alanine (L-Ala) | 0.8 mM |

**Table S2** Strains and plasmids used in monoterpene production

| **Strains** | **Description** | **Reference** |
| --- | --- | --- |
| JPUB_019980 | *P. putida* KT2440 with pBbB5k-MTSA-T1-MK_sc_-PMK-PMD_sc_-idi-T1-trGPPS-LS | This study |
| JPUB_019984 | *P. putida* KT2440 with pBbB5k-MTSA-T1-MK_sc_-PMK-PMD_sc_-idi-T1-trGPPS-CS | This study |
| **Plasmids** | **Description** | **Reference** |
| JPUB_019930 | pBbB5k-MTSA-T1-MK_sc_-PMK-PMD_sc_-idi-T1-trGPPS-LS | This study |
| JPUB_019932 | pBbB5k-MTSA-T1-MK_sc_-PMK-PMD_sc_-idi-T1-trGPPS-CS | This study |

**Fig. S1** Comparison of carbon flux distribution between *E. coli* and *P. putida*. The published ^13^C-metabolic flux data was used to compare the difference in carbon flux. Number in black, carbon flux of *E. coli* [2]; Number in red, carbon flux of *P. putida* [3]. PEP, phosphoenolpyruvate; Pry, pyruvate; AcCoA, acetyl-CoA; Cit, citrate, AKG, 2-ketoglutarate; Mal, malate; OAA, oxaloacetate.

**
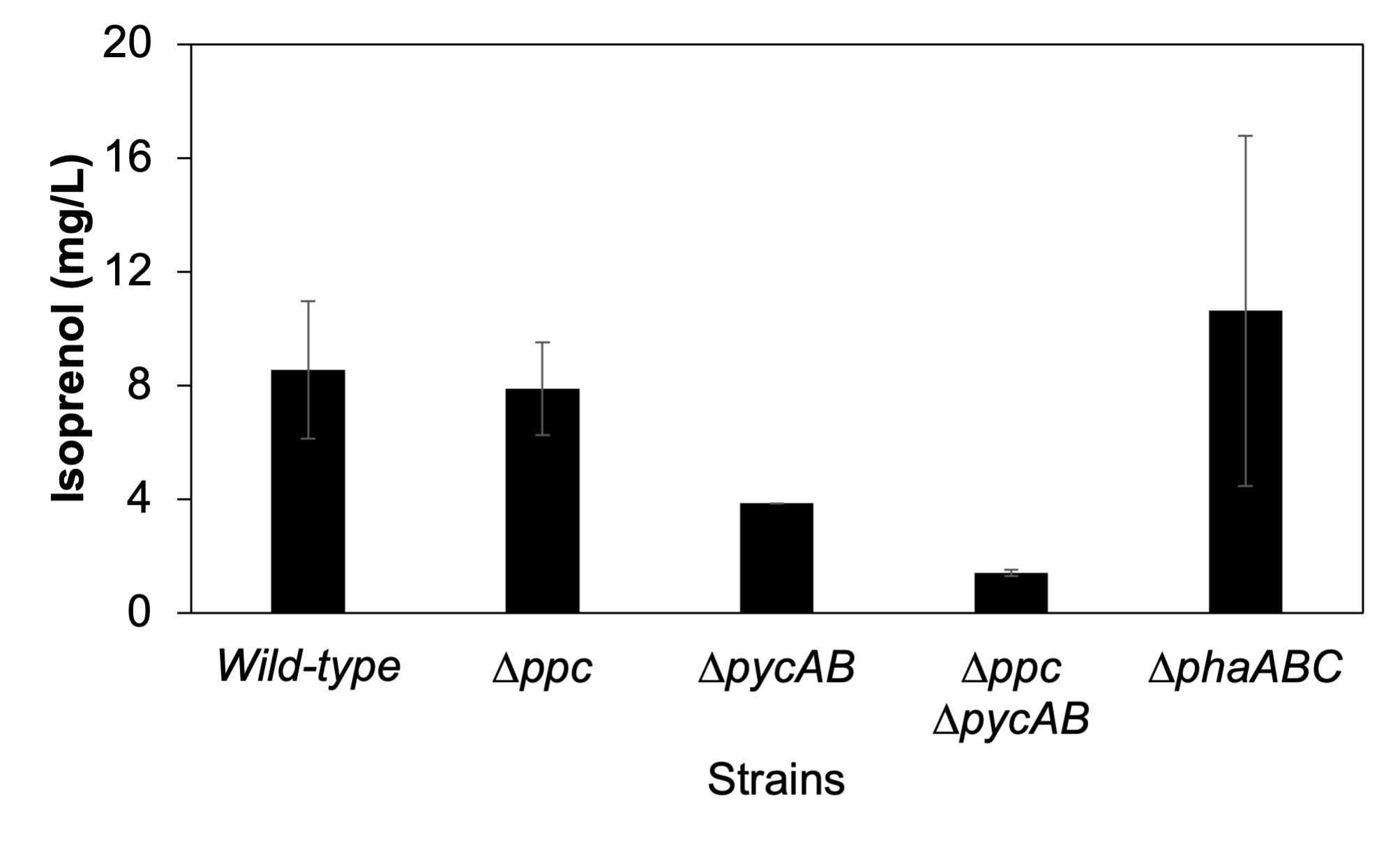
**

**Fig. S2** Comparison of isoprenol production with *P. putida* gene-knockout strains bearing JPUB_019918 plasmid in a microtiter plate. A 24-well microtiter plate was used for screening isoprenol production using the EZ-rich medium containing 1% glucose. Data was shown after 48-hour production. Error bars indicate one standard deviation of triplicates.


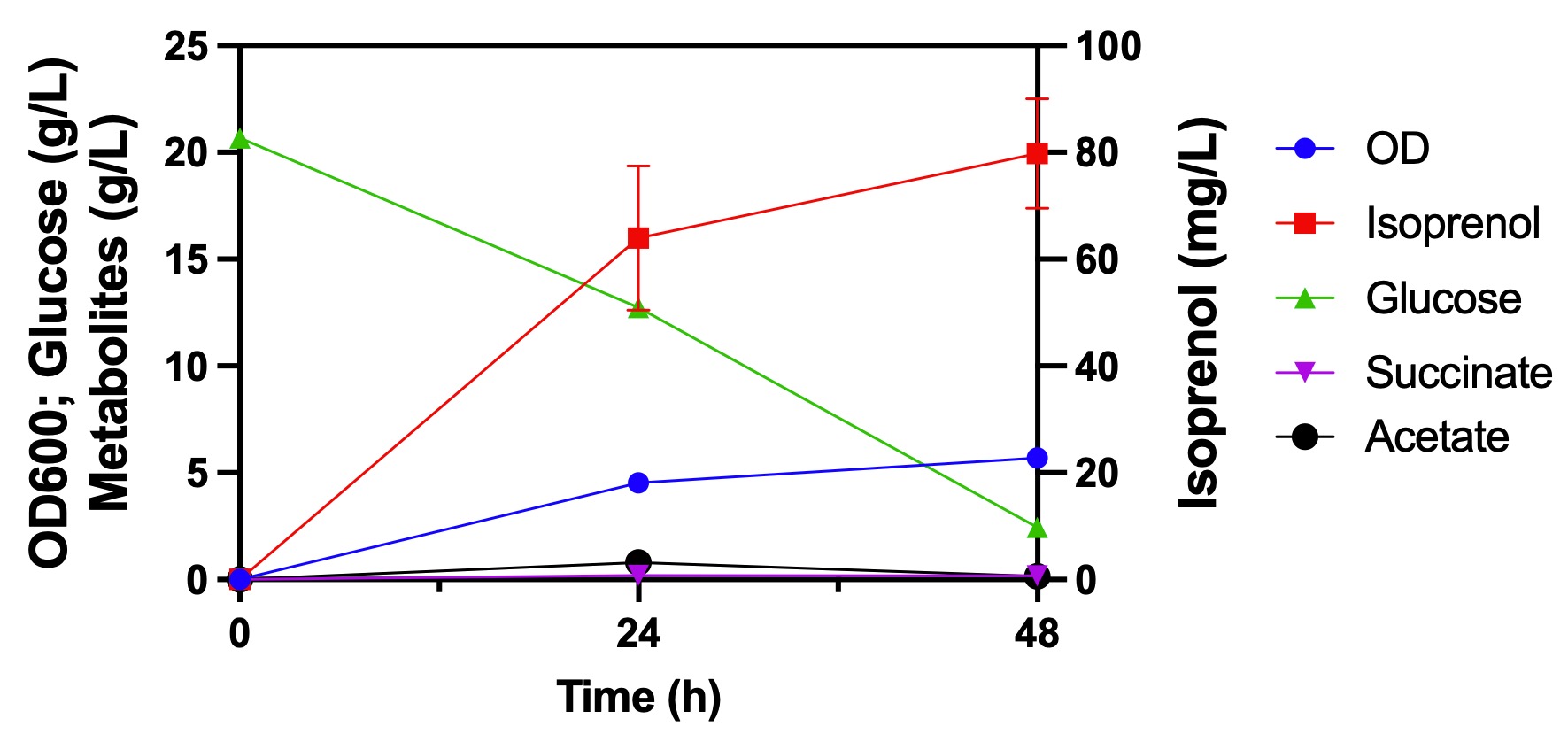


**Fig. S3** Isoprenol production with *P. putida* Δ*phaABC* strain (JPUB_019964) bearing JPUB_019925 plasmid in a shake flask using EZ-rich medium containing 2% glucose. Error bars indicate one standard deviation of triplicates.


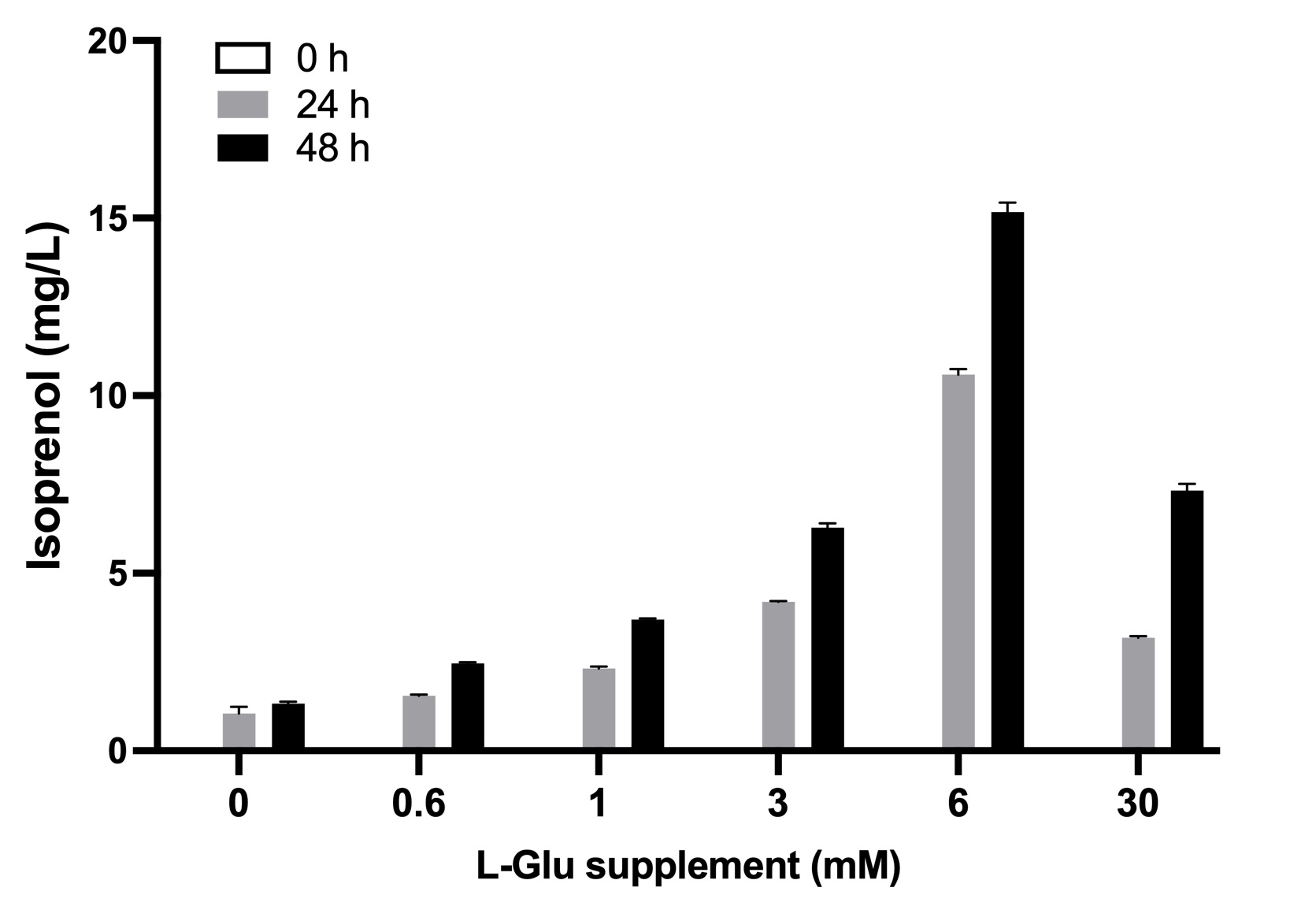


**Fig. S4** Isoprenol production by JPUB_019977 strain in M9 minimal medium supplemented with different concentrations of L-Glu. Error bars indicate one standard deviation of triplicates.


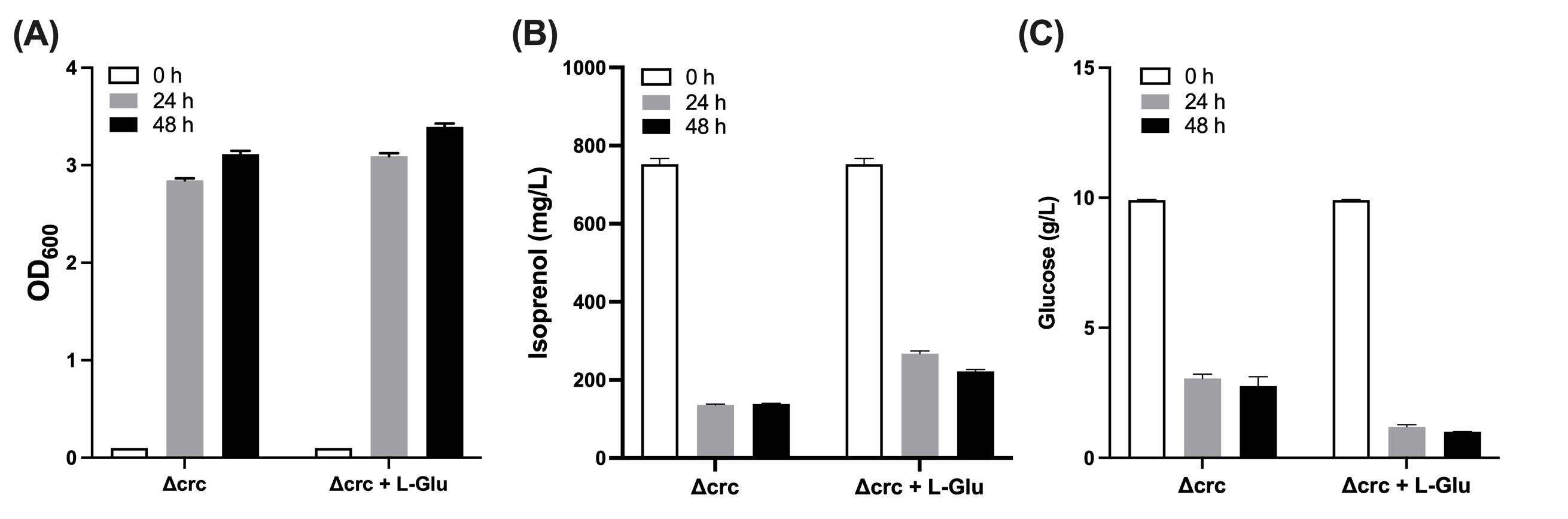


**Fig. S5** Investigation of isoprenol consumption for *P. putida* Δ*crc* strain (JPUB_019978). Isoprenol consumption was performed in M9 minimal medium containing 1% glucose with or without 0.6 mM L-Glu supplement. (A) Cell growth represented by OD_600_; (B) Isoprenol consumption; (C) Glucose consumption. Error bars indicate one standard deviation of triplicates.


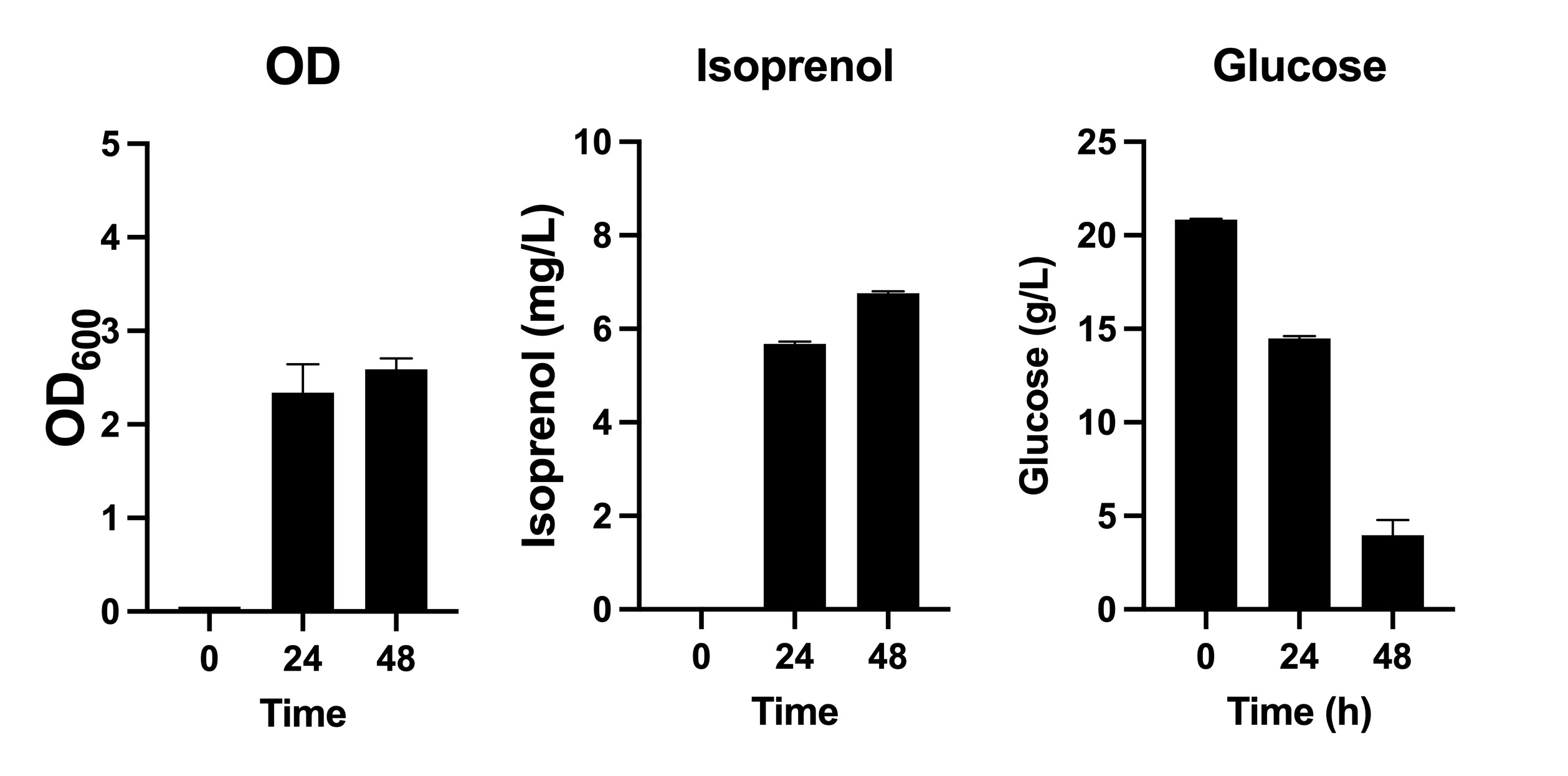


**Fig. S6** Isoprenol production with *crc* overexpression by *P. putida* Δ*phaABC* strain (JPUB_019964) with plasmid JPUB_019949 from 2% glucose. Error bars indicate one standard deviation of triplicates.

**
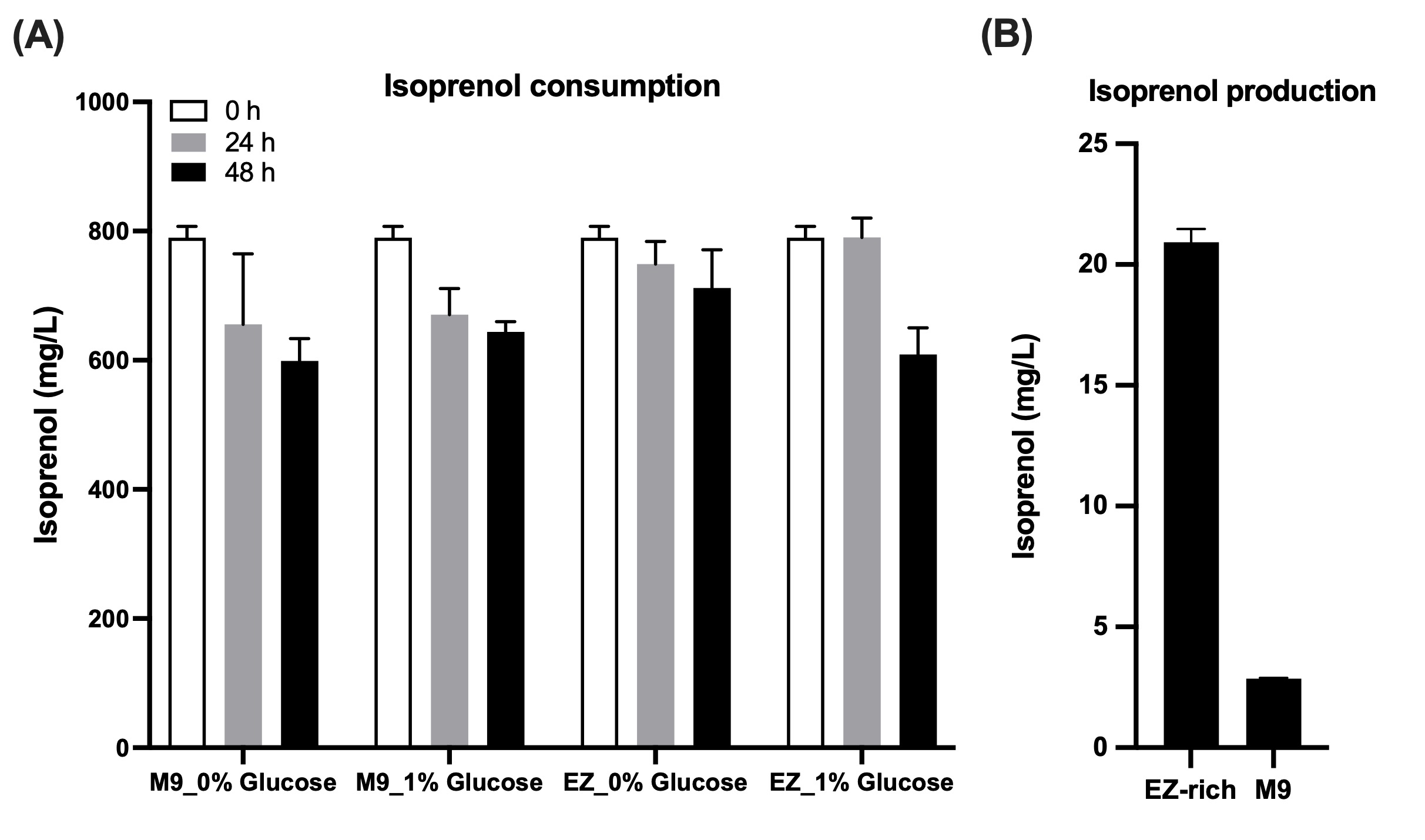
**

**Fig. S7** Isoprenol consumption and production in *P. putida* Δ*phaABC* ΔPP_2675 strain (JPUB_019965). (A) Isoprenol consumption in M9 minimal medium and EZ-rich medium containing 1% glucose or no glucose, respectively. Results verified that the deletion of PP_2675 prevented isoprenol from self-consumption. (B) Isoprenol production in M9 minimal medium and EZ-rich medium containing 2% glucose using JPUB_019925 plasmid. The deletion of PP_2675 did not improve isoprenol titer. Error bars indicate one standard deviation of triplicates.

**
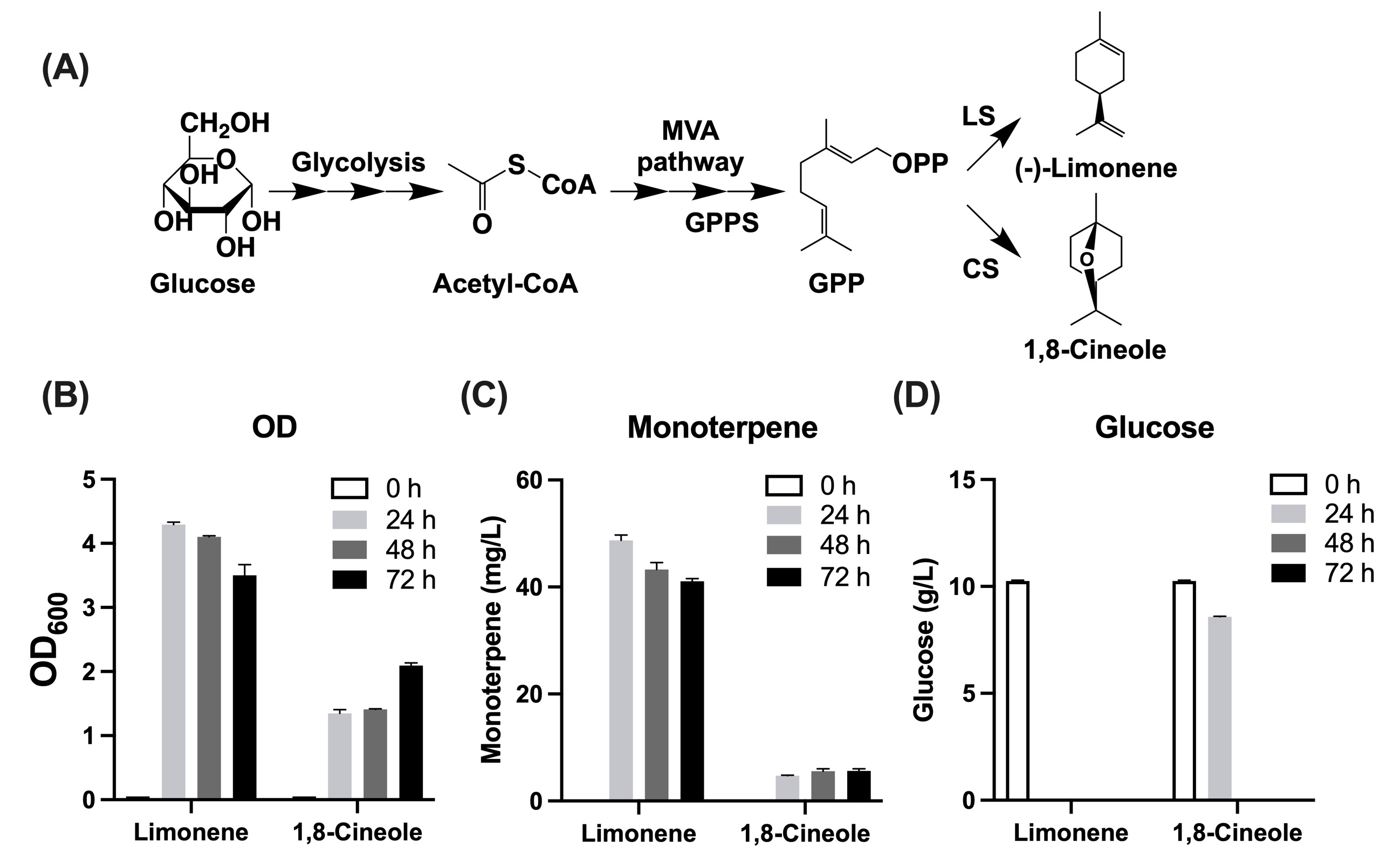
**

**Fig. S8** Production of monoterpene in the engineered *P. putida*. (A) Monoterpene synthesis pathway from glucose. (B-D) Production results by the engineered *P. putida* strains from 1% glucose. (B) Cell growth. The cell growth of the 1,8-cineole-producing strain was slower than the limonene-producing strain (C) Monoterpene production. The highest limonene production was 49 mg/L after 24 h, and the highest 1,8-cineole production was 6 mg/L after 72 h. (D) Glucose consumption. The 1,8-cineole-producing strain remained at 8.6 g/L glucose after 24 h, which might explain the slower cell growth. Error bars indicate one standard deviation of triplicates. GPP, geranyl diphosphate; GPPS, geranyl diphosphate synthase; LS, limonene synthase from *Mentha spicata*. CS, 1,8-cineole synthase from *Streptomyces clavuligerus*. Error bars indicate one standard deviation of triplicates.

**
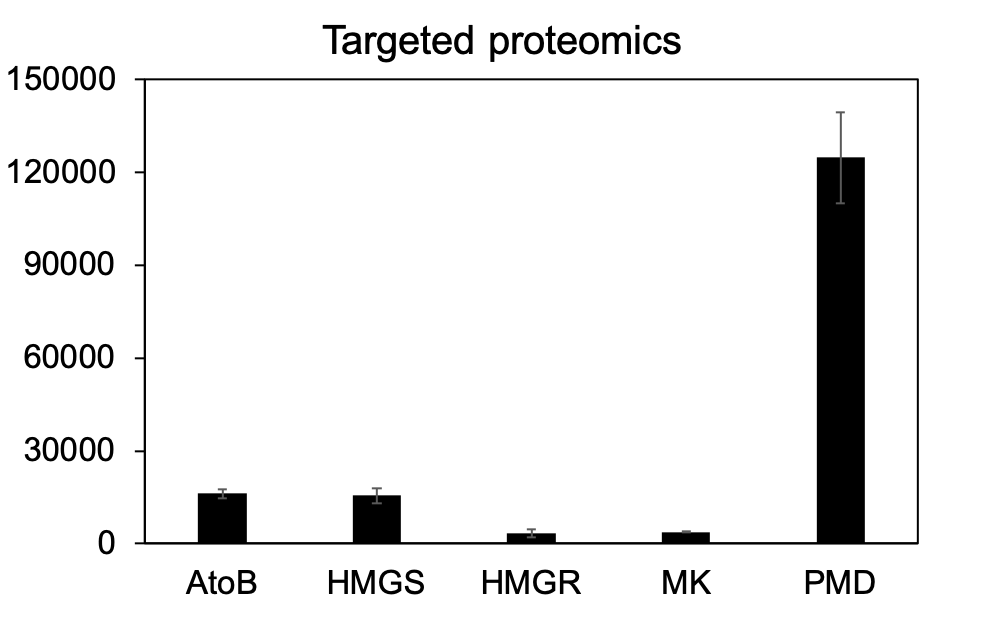
**

**Fig. S9** Targeted proteomics of IPP-bypass MVA pathway in isoprenol production. Targeted proteomics was conducted as described previously [4]. The results confirmed heterologous gene expression of the MVA pathway in *P. putida* strain JPUB_019968 during isoprenol production. Error bars indicate one standard deviation of triplicates.

**References**

1. Wang X, Pereira JH, Tsutakawa S, Fang X, Adams PD, Mukhopadhyay A, et al. Efficient production of oxidized terpenoids via engineering fusion proteins of terpene synthase and cytochrome P450. Metabolic Engineering. 2021;64:41–51.

2. Gonzalez JE, Long CP, Antoniewicz MR. Comprehensive analysis of glucose and xylose metabolism in Escherichia coli under aerobic and anaerobic conditions by 13C metabolic flux analysis. Metabolic Engineering. 2017;39:9–18.

3. Nikel PI, Chavarría M, Fuhrer T, Sauer U, de Lorenzo V. Pseudomonas putida KT2440 Strain Metabolizes Glucose through a Cycle Formed by Enzymes of the Entner-Doudoroff, Embden-Meyerhof-Parnas, and Pentose Phosphate Pathways. Journal of Biological Chemistry. 2015;290:25920–32.

4. Mendez-Perez D, Alonso-Gutierrez J, Hu Q, Molinas M, Baidoo EEK, Wang G, et al. Production of jet fuel precursor monoterpenoids from engineered Escherichia coli. Biotechnology and Bioengineering. John Wiley & Sons, Ltd; 2017;114:1703–12.
